# Integrative metabolomics and transcriptomics signatures of clinical tolerance to *Plasmodium vivax* reveal activation of innate cell immunity and T cell signaling

**DOI:** 10.1016/j.redox.2018.04.011

**Published:** 2018-04-11

**Authors:** Luiz G. Gardinassi, Myriam Arévalo-Herrera, Sócrates Herrera, Regina J. Cordy, ViLinh Tran, Matthew R. Smith, Michelle S. Johnson, Balu Chacko, Ken H. Liu, Victor M. Darley-Usmar, Young-Mi Go, MaHPIC Consortium, Dean P. Jones, Mary R. Galinski, Shuzhao Li

**Affiliations:** a Department of Medicine, School of Medicine, Emory University, 615 Michael Street, Atlanta, GA 30322-1047, USA; b Malaria Vaccine and Drug Development Center (MVDC), Cali, Colombia; c Faculty of Health, Universidad del Valle, Cali, Colombia; d Caucaseco Scientific Research Center, Cali, Colombia; e International Center for Malaria Research, Education and Development, Emory Vaccine Center, Yerkes National Primate Research Center, Emory University, Atlanta, GA, USA; f Department of Pathology and Mitochondrial Medicine Laboratory, University of Alabama at Birmingham, Birmingham, AL, USA

**Keywords:** Malaria, *Plasmodium vivax*, Tolerance, Metabolomics, Transcriptomics, Integration, Platelets, Immunity

## Abstract

Almost invariably, humans become ill during primary infections with malaria parasites which is a pathology associated with oxidative stress and perturbations in metabolism. Importantly, repetitive exposure to *Plasmodium* results in asymptomatic infections, which is a condition defined as clinical tolerance. Integration of transcriptomics and metabolomics data provides a powerful way to investigate complex disease processes involving oxidative stress, energy metabolism and immune cell activation. We used metabolomics and transcriptomics to investigate the different clinical outcomes in a *P. vivax* controlled human malaria infection trial. At baseline, the naïve and semi-immune subjects differed in the expression of interferon related genes, neutrophil and B cell signatures that progressed with distinct kinetics after infection. Metabolomics data indicated differences in amino acid pathways and lipid metabolism between the two groups. Top pathways during the course of infection included methionine and cysteine metabolism, fatty acid metabolism and urea cycle. There is also evidence for the activation of lipoxygenase, cyclooxygenase and non-specific lipid peroxidation products in the semi-immune group. The integration of transcriptomics and metabolomics revealed concerted molecular events triggered by the infection, notably involving platelet activation, innate immunity and T cell signaling. Additional experiment confirmed that the metabolites associated with platelet activation genes were indeed enriched in the platelet metabolome.

## Highlights

• *Plasmodium vivax* infection induces significant change in blood metabolomics.
• Naïve and semi-immune subjects exhibit different molecular profiles.
• Network integration of metabolites/genes hinges on innate activation, chemokines and T cell signaling.
• Involvement of platelet activation is confirmed by platelet metabolomics.

## Graphical abstract

**Figure:**
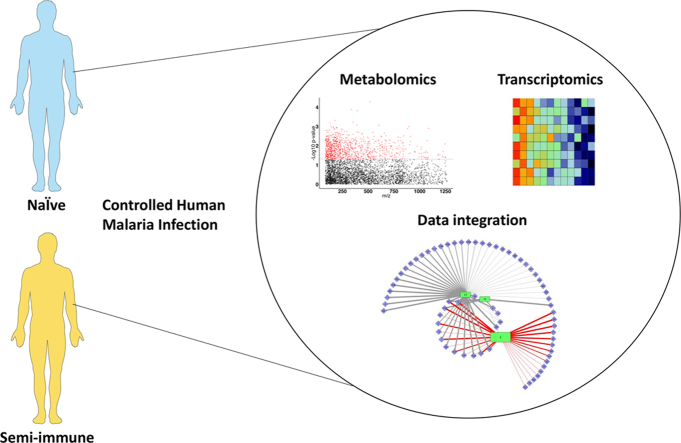

## 1 Introduction

Infections with *Plasmodium vivax* constitute a major public health problem worldwide. *P. vivax* accounted for 41% of estimated malaria cases reported in 2015 outside the African continent [1]. *P. vivax* malaria is characterized by a febrile illness, which may develop into severe symptoms and fatal complications [2]. However, depending on the history of exposure to the parasite, some infections can also remain asymptomatic [3], [4]. The molecular mechanisms underlying host responses to *P. vivax* are poorly understood. Controlled human malaria infection (CHMI) trials have become a critically important research tool, and have been used to evaluate the immunization efficacy for *P. vivax* [5], [6], [7]. A recent CHMI trial confirmed that previous exposure to *P. vivax* leads to reduced symptoms such as fever and headache [8]. Antibodies from semi-immune individuals reacted to a larger repertoire of *P. vivax* antigens before infection [9], however this was insufficient to control parasite growth after sporozoite challenge [8]. Moreover, symptomatic semi-immune individuals exhibited similar antibody kinetics to that of naïve. Clinical tolerance was associated with antibody reactivity to a smaller subset of antigens [9].

In recent years, important technical developments have emerged allowing the investigation of the human immune responses via high-throughput data [10], [11], [12], [13], [14], [15], [16]. These include analyses of human samples using transcriptomics, proteomics, metabolomics, lipidomics and single-cell profiling. Because the human immune system has important differences from animal models, and good animal models are not always available, these studies lead to a detailed understanding of human immunity which is otherwise inaccessible. Metabolomics is global profiling of small molecules in tissues, cells and biological fluids [13]. It captures a “snapshot” of the activity of metabolic processes and molecular phenotypes. Small molecules (metabolites and lipids) not only serve the metabolic need of growth and survival, but also regulate functions of immune cells and systemic signals in infection and inflammation [17], [18], [19], [20]. It has been reported that *P. falciparum* malaria results in altered abundance of plasma metabolites involved in lipid [21], energy [22], and amino acid metabolism [23]. A metabolomics investigation of *P. vivax* infected patients revealed that their parasite load was associated with heme metabolism and lipid pathways [24].

In this study, we describe the plasma metabolomes from distinct clinical outcomes of a *P. vivax* CHMI trial, and provide novel insights into associated blood transcriptomes. These are facilitated by our recent development in bioinformatics tools [16], [25], [26]. Furthermore, the integration of metabolomics and transcriptomics in *P. vivax* CHMI revealed metabolic processes that were significantly associated with oxidative stress and immunological modalities.

## 2 Methods

### 2.1 Ethics statement and clinical trial

The clinical study was conducted at the Malaria Vaccine and Drug Development Center (CIV, Cali) [8]. It was approved by the Institutional Review Boards (IRB) at the CIV and Centro Médico Imbanaco, Cali (Trial Accession #NCT01585077) and the University of Alabama at Birmingham Institutional Review Board (Protocol #X110718014). Written informed consent was obtained at enrollment. Among one-hundred individuals assessed for eligibility at CIV, sixty-nine did not meet inclusion criteria, while fifteen declined to participate. Exclusion criteria included pregnancy; glucose-6-phosphate dehydrogenase (G6PDH) deficiency; positive reactivity for syphilis, HIV, Chagas disease, HTLV 1–2, hepatitis (B – C); or any condition that could increase the risk of adverse outcomes [27], [28]. The remaining individuals (n = 16) were allocated into two groups according to the status of previous exposure to *P. vivax*. Naïve subjects (n = 7) were recruited in Cali (Colombia), a city in which malaria is not endemic. Semi-immune subjects (n = 9) were recruited in Buenaventura, an endemic city for malaria located on the Pacific Coast. The study was exploratory and the sample size was not based on pre-defined effect size but limited by enrollment. Subjects were healthy male and female adults, Duffy-positive (Fy+), 18–45 years of age. The degree of immunity to *P. vivax* was assessed by clinical history and presence of antibodies against *P. vivax* blood stages. *Anopheles albimanus* mosquitoes were reared and infected at the MVDC insectary in Cali, and sporozoite challenge of all subjects was conducted on the same day. Subjects were exposed to bites of 2–4 infected mosquitos from the same batch. The subjects were monitored daily for clinical manifestations and patent parasitemia. One of the subjects did not develop parasitemia and was excluded from the study. *P. vivax* infection in challenged subjects was confirmed with thick blood smears (TBS), and retrospectively by RT-qPCR. On the day of parasite detection by TBS, subjects were treated orally with curative doses of chloroquine (1500 mg provided in three doses) and primaquine (30 mg administered once a day for 14 days) [8]. Plasma samples were collected before infection (baseline), on the day of positive blood smear (diagnosis), and three-weeks after treatment.

### 2.2 Metabolomics analysis and data processing

Liquid chromatography-mass spectrometry (LC-MS) analyses were performed as described [16], [29], [30]. Briefly, acetonitrile (2:1, v/v) was added to 65 µL of plasma and centrifuged at 14,000 g for 10 min at 4 °C to remove proteins. The supernatant was transferred to an auto sampler vial for LC-MS, using a High Field Q Exactive mass spectrometer (Thermo Fisher), coupled with HILIC liquid chromatography. Mass spectral data was acquired with positive electrospray ionization and the full scan of mass-to-charge ratio (*m/z*) ranged from 85 to 1275 at a resolution of 120,000. Each sample was run in triplicate in batches of 20 samples. Peak detection, noise filtering, *m/z*, and retention time alignment, and feature quantification were performed with apLCMS [31] and xMSanalyzer [32]. Each metabolite feature is defined by *m/z* and retention time, with intensity values associated with each replicate. Data were averaged among replicates, log2 transformed and normalized by the mean. Only features detected in more than 75% of all samples (4236) were used in further analysis. The reporting of metabolite annotation adheres to the five confirmation levels in metabolomics literature [33], [34]. Level 1 annotation applies to the metabolites confirmed by matching both *m/z* (mass accuracy under 10 ppm) and retention time to that of authenticated chemical standards, previously characterized in our laboratory (Supplemental Table 1). Additional putative annotation was performed by *m/z* matching to KEGG database (mass accuracy under 10 ppm – annotation level 3) [35]. The *mummichog* software (version 1.0.7) was used for metabolic pathway analysis (mass accuracy under 10 ppm) [25]. The raw metabolomics data have been made publicly available in the MetaboLights repository (https://www.ebi.ac.uk/metabolights/, study ID MTBLS665). A list of all publicly available MaHPIC datasets is at http://plasmodb.org/plasmo/mahpic.jsp.

### 2.3 Platelet isolation

Collection of human platelets was approved by the University of Alabama at Birmingham Institutional Review Board, and performed as described [36]. Briefly, platelet-rich plasma, obtained from individual donors from the blood bank at the University of Alabama at Birmingham, was centrifuged at 1500 g for 10 min, and washed with PBS containing prostaglandin I2 (1 µg/ml). Platelets were diluted in DMEM assay buffer (DMEM with 1 mM pyruvate, 5.5 mM D-glucose, 4 mM L-glutamine, pH 7.4), and incubated for 3 h at 37 °C. After washing with cold PBS, platelets were spiked with internal isotope standards, and distributed to a final concentration of 300 × 10^6^/150 µL of acetonitrile. After incubation on ice for 15 min, proteins were removed by centrifugation at 13,000 rpm for 10 min at 4 °C. Supernatants were stored at − 80 °C. LC-MS analysis was carried out as described above. For comparisons with plasma metabolomics, platelet's metabolite features were averaged across samples from individual donors (n = 5), and the 200 most abundant features were used as platelet-enriched metabolites. Hypergeometric test was performed with the *phyper* function in R, to evaluate accurate plasma metabolite over-representation in platelet-enriched metabolites.

### 2.4 Whole blood transcriptional analyses

Blood collection, RNA extraction and sequencing, and raw data pre-processing pipeline were described previously [37]. Data are available at the Gene Expression Omnibus repository, accession number GSE67184. Gene set enrichment analysis (GSEA) [38] was performed with 1000 permutations and weighted enrichment statistic. Blood Transcription Modules (BTMs) were used as gene sets [26]. Significant enrichment was determined by a false discovery rate (FDR) < 0.05. BTMs were also used for dimension reduction, in reporting the transcriptomic findings and in the integration with metabolomics. The transcriptomics data were mapped to 281 BTMs based on the coverage of sequencing, and module activity was taken as the mean expression value of member genes.

### 2.5 Statistical and bioinformatics analyses

Both metabolomics and transcriptomics data were log2 transformed so that they followed normal distribution. Repeated measures ANOVA was used to evaluate differences between time points of CHMI trial. *Limma* moderated *t*-statistic was used to evaluate differences in categorical comparisons (naïve vs semi-immune subjects). Euclidian distance and Ward linkage algorithm was used for the hierarchical clustering. P-values less than 0.05 were considered significant. False discovery rate (FDR) was calculated using the Benjamini–Hochberg method. Categorical comparisons were performed with normalized intensity values at baseline time point. At diagnosis and post-treatment time points, comparisons were performed with baseline subtracted intensity values to remove individual confounding factors, indicated as diag./basel. or post-treat./basel. Variance in each group was similar when individual genes/metabolites were plotted. In the box plots and line plots, mean values are shown and error bars are based on standard deviations.

Integration of metabolomics and transcriptomics data, at baseline and diagnosis/baseline, was performed similarly as previously described [16]. Briefly, the transcriptomics data were collapsed to BTMs, and module activity scores were taken as the mean value of member genes [26]. Next, unsupervised hierarchical clustering was applied to BTMs’ activities to generate BTM clusters. Metabolite features were clustered similarly, but with a modified metrics to enforce close retention time within clusters. Associations between BTM clusters and metabolite clusters were estimated by partial least square (PLS) regression. The significance of associations was computed on 1 million permutations, resampling both features and sample labels. The resulting networks were visualized using Cytoscape 3.4.0 (http://cytoscape.org).

## 3 Results

**Fig. 1:**
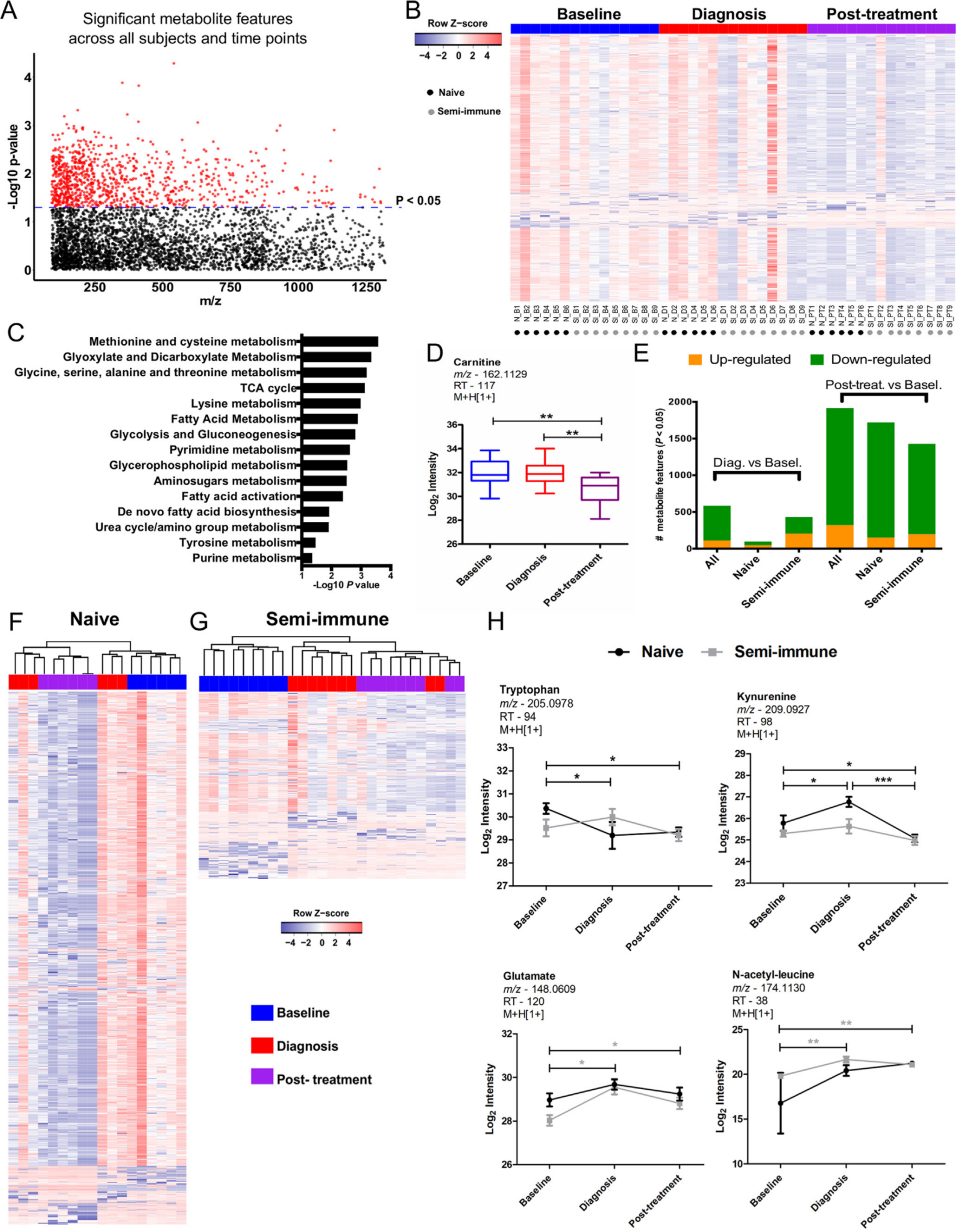
**Metabolomic signatures during the*****P. vivax*****CHMI trial.***A*, Manhattan plot for significant metabolite features (964 at p < 0.05; 0 at FDR < 0.1; colored in red) in time course analysis for all subjects. *B*, One-way hierarchical clustering based on significant metabolite features in time course analysis for all subjects. *C*, Metabolic pathways enriched by significant metabolite features in time course analysis for all subjects. *D*, Boxplot for carnitine's abundance kinetics in time course analysis for all subjects. *E*, Differential abundance of metabolite features compared to baseline. *F*-*G*, Two-way hierarchical clustering based on significant metabolite features in time course analysis for naïve (1577 at p < 0.05; 454 at FDR < 0.1) or semi-immune (688 at p < 0.05; 180 at FDR < 0.1) subjects. *H*, Abundance kinetics for plasma tryptophan, kynurenine, glutamate and N-acetyl-leucine. Significant metabolite features were identified by ANOVA with repeated measures. Tukey's multiple comparisons test was used in additional statistics. Significance levels are shown as * , p < 0.05; **, p < 0.01; ***, p < 0.001. (For interpretation of the references to color in this figure legend, the reader is referred to the web version of this article)

**Fig. 2:**
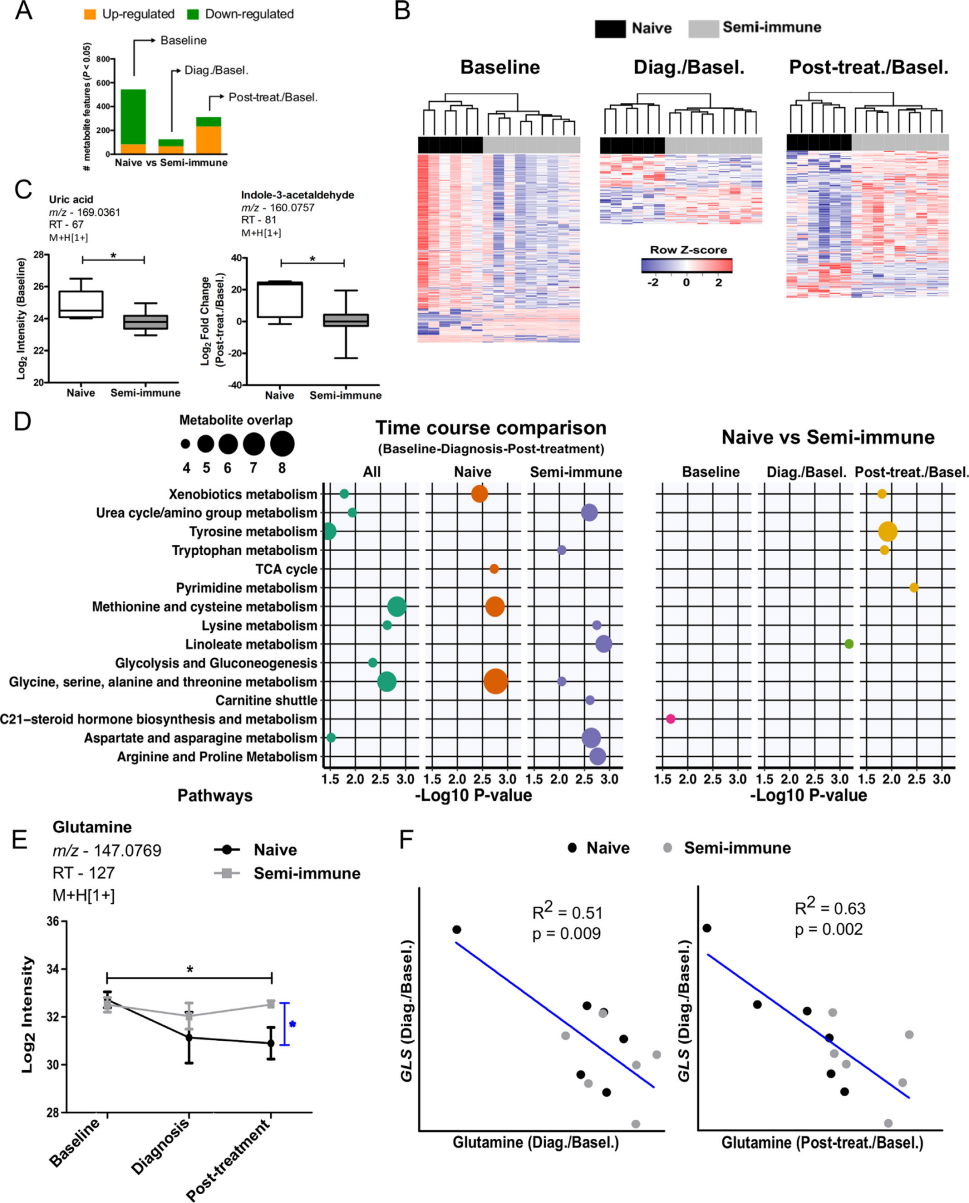
**Differential metabolite abundance between naïve and semi-immune subjects.***A*, Significant metabolite features differing between naïve and semi-immune subjects. Normalized intensity values were used to identify significant features at baseline. Baseline normalized intensity values were used to identify significant features at diagnosis and post-treatment time points. *Limma* moderated *t-*statistic was used. *B*, Two-way hierarchical clustering based on differentially abundant metabolite features between naïve and semi-immune subjects at baseline, diagnosis/baseline and post-treatment/baseline. *C*, Boxplots for uric acid (baseline) or indole-3-acetaldehyde (post-treatment/baseline). Significance level is shown as *, p < 0.05. *D*, Summary of metabolic pathways enriched by significant metabolite features. *Mummichog* software was used for pathway enrichment using the top 200 most significant metabolite features for each statistical comparison between time points or immune status. Only pathways represented by at least four metabolite features and enriched at p < 0.05 are shown. *E,* Abundance kinetics for plasma glutamine. Significant metabolite features were identified by ANOVA with repeated measures. Tukey's multiple comparisons test was used in additional statistics. Significance levels are shown as *, p < 0.05. *F*, Correlation between plasma glutamine and blood cell glutaminase (*GLS*) expression assayed by RNA-seq.

**Fig. 3:**
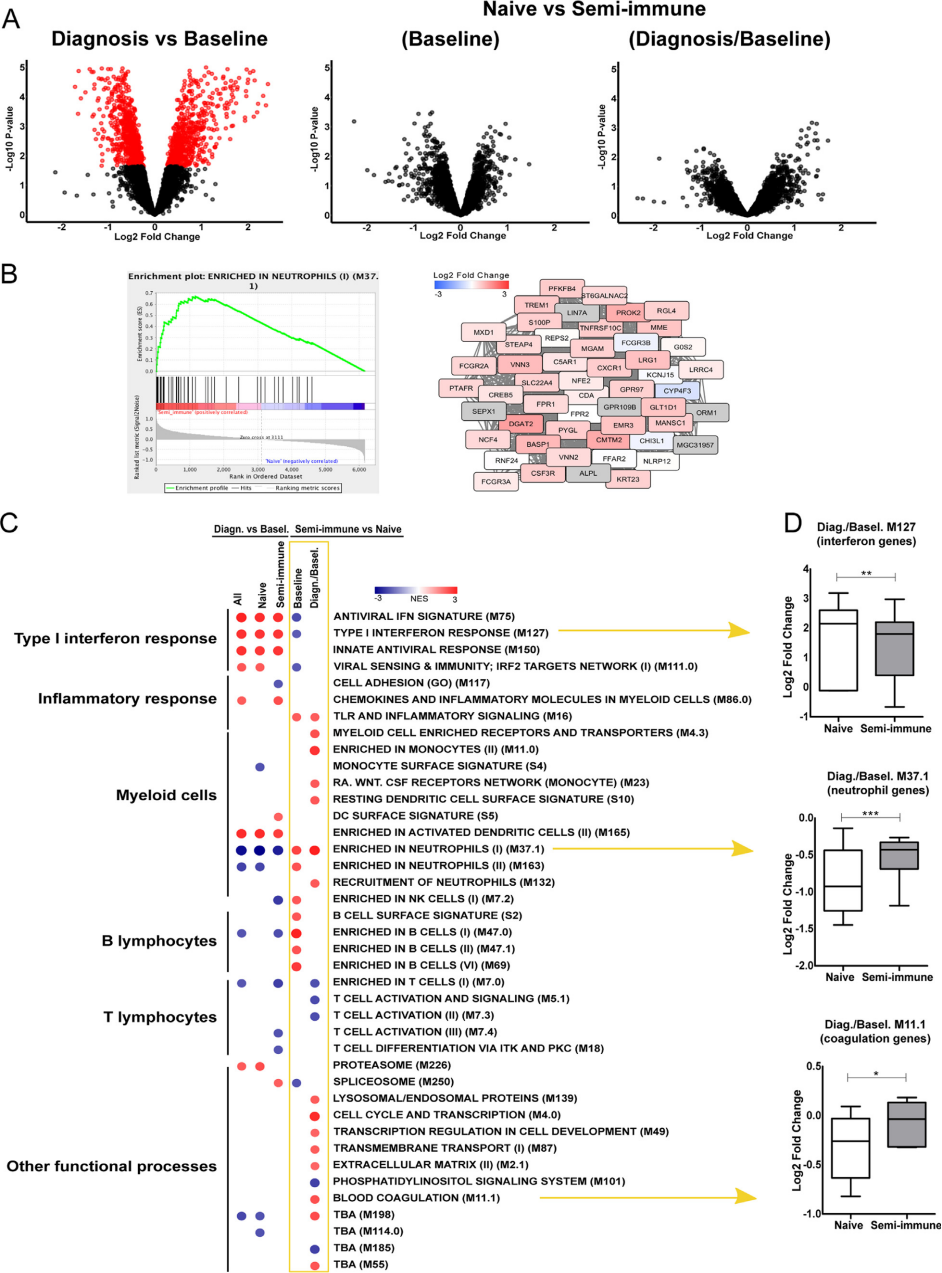
**Blood Transcription Modules (BTM) analysis of*****P. vivax*****CHMI trial.** Blood transcriptomes of six naïve and six semi-immune subjects were described previously. *A*, Gene-level analyses for all subjects between diagnosis and baseline levels; between naïve and semi-immune subjects at baseline, and at the time of *P. vivax* malaria diagnosis. Genes selected at FDR < 0.1 are colored in red. *B*, Representative gene set ranking plot and, network plot for BTM annotated as Enriched in Neutrophils (II) (M37.1). It shows the comparison between semi-immune and naïve subjects at the time of diagnosis. Blue to red scale in the network corresponds to gene expression fold changes of semi-immune/naïve subjects. Genes that were not measured in this experiment are colored in gray. *C*, Summary of BTMs associated with transcriptional profiles according to each statistical comparison. Comparison between semi-immune and naïve subjects at the time of diagnosis was based on baseline normalized intensities. Gene set enrichment analysis was used to identify significant associations at FDR < 0.05. The blue to red scale indicates negative or positive associations based on normalized enrichment scores (NES). *D,* Boxplots of highlighted BTMs comprising interferon genes (M127), neutrophil genes (M37.10, and blood coagulation genes (M11.1). Significance levels are shown as *, p < 0.05; **, p < 0.01; ***, p < 0.001. (For interpretation of the references to color in this figure legend, the reader is referred to the web version of this article).

**Fig. 4:**
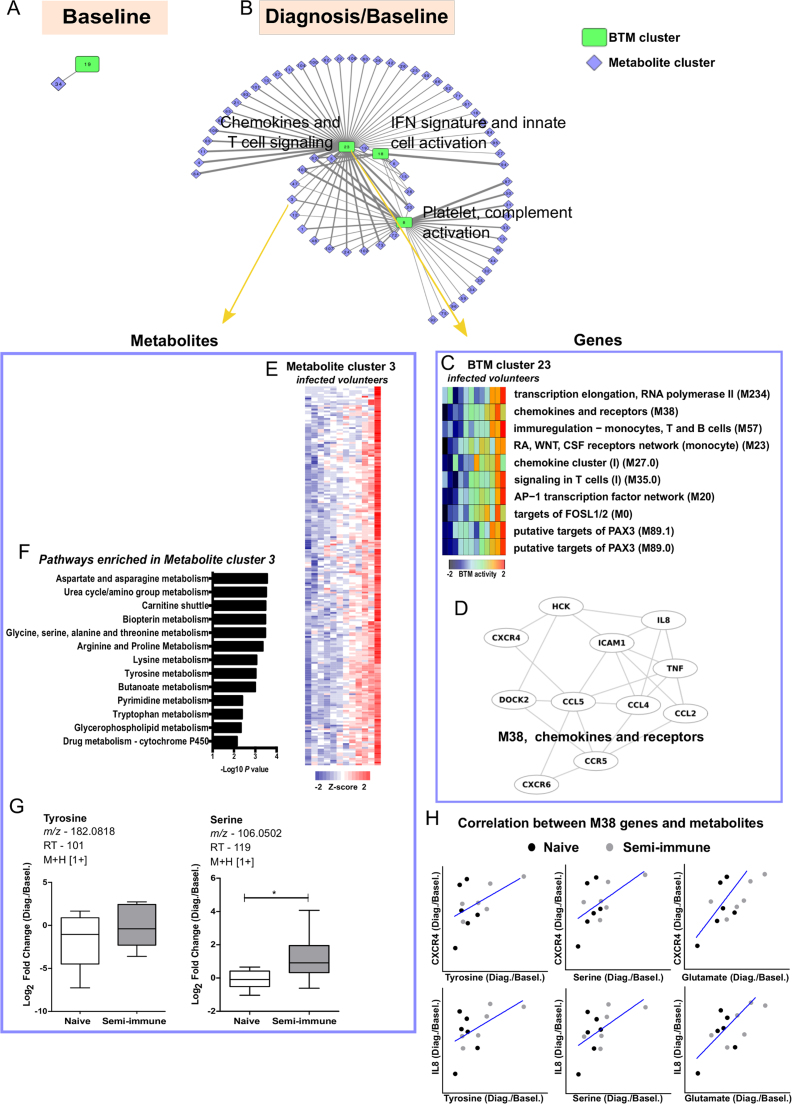
**Integrated metabolomic and transcriptomic response network of*****P. vivax*****infection.** Each node represents a sub-network of metabolomics (Metabolite cluster) or transcriptomics (BTM cluster) data. Links between nodes represent significant associations using partial least square regression and permutation test. *A,* Significant association at baseline. *B*, Significant associations at the time of *P. vivax* malaria diagnosis (baseline normalized intensity values were used). *C*, Heat map of BTMs’ activity for each *P. vivax* infected subject, included in BTM cluster 23. *D*, The BTM module 38 comprises chemokines and receptors genes. *E,* Heat map of metabolites for each *P. vivax* infected subject, included in Metabolite cluster 3. *F*, Metabolic pathways enriched in Metabolite cluster 3. *Mummichog* software was used to evaluate metabolic pathways. *G*, Boxplots for tyrosine and serine at diagnosis/baseline. Significance level is shown as *, p < 0.05. *H,* Correlation between plasma tyrosine, serine and glutamate with M38 member gene *CXCR4* and *IL8* expression assayed by RNA-seq.

**Fig. 5:**
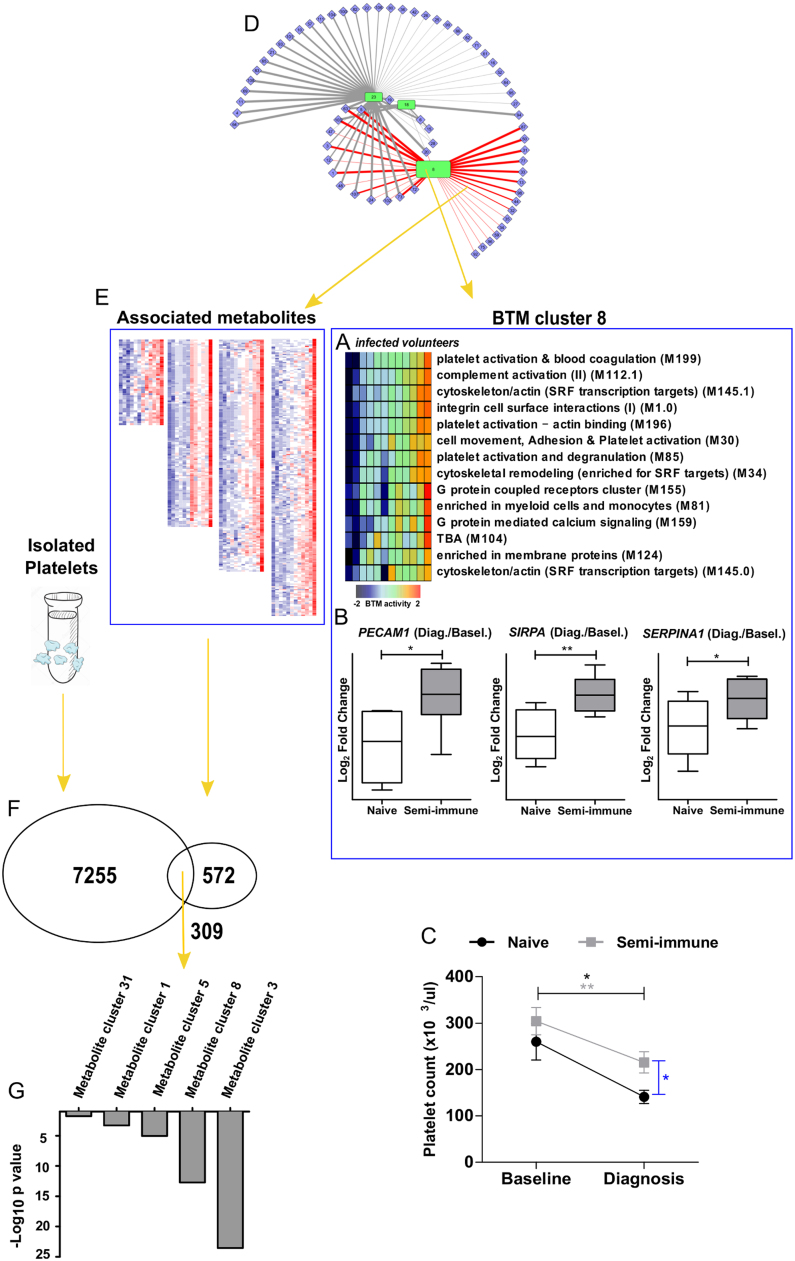
**Molecular signatures of platelet activation in*****P. vivax*****infection.***A,* Heat map of BTMs’ activity for each *P. vivax* infected subject, included in BTM cluster 8 (Platelet, complement activation) identified by integrative metabolomics and transcriptomics as described in Fig. 4B. *B*, Boxplots of platelet activation genes *PECAM1, SIRPA, SERPINA1* expression assayed by RNA-seq. *C*, Platelet counts from whole blood at baseline and diagnosis time points. Significance levels are shown as * , p < 0.05; **, p < 0.01. *D,* Highlighted associations between metabolites and gene cluster 8, as described in Fig. 4B. *E*, Metabolite clusters associated with gene cluster 8 exemplified as heatmaps. *F*, Venn diagram showing common *m/z* peaks detected in platelets derived from healthy donors and plasma metabolite clusters associated with gene cluster 8. *G,* Metabolite cluster *m/z* peak over-representation in platelet-enriched metabolites.

This CHMI study included two groups of participants: one group naïve and the other semi-immune to *P. vivax* infection and which was shown to be more tolerant to symptoms of *P. vivax* infection [8]. The goal of this study is to delineate the molecular differences between the naïve group and the semi-immune group. To this end, we first report the metabolomic changes during the time course of infection (Fig. 1), then compare the two groups by their plasma metabolomics (Fig. 2) and blood transcriptomics (Fig. 3). The integration of metabolomics and transcriptomics revealed significant associations induced by the infection (Fig. 4), and we further validate the platelet activation pathway suggested by the integrative analysis (Fig. 5).

### 3.1 Plasma metabolomic signatures along the course of *Plasmodium vivax* CHMI

**Fig. S1:**
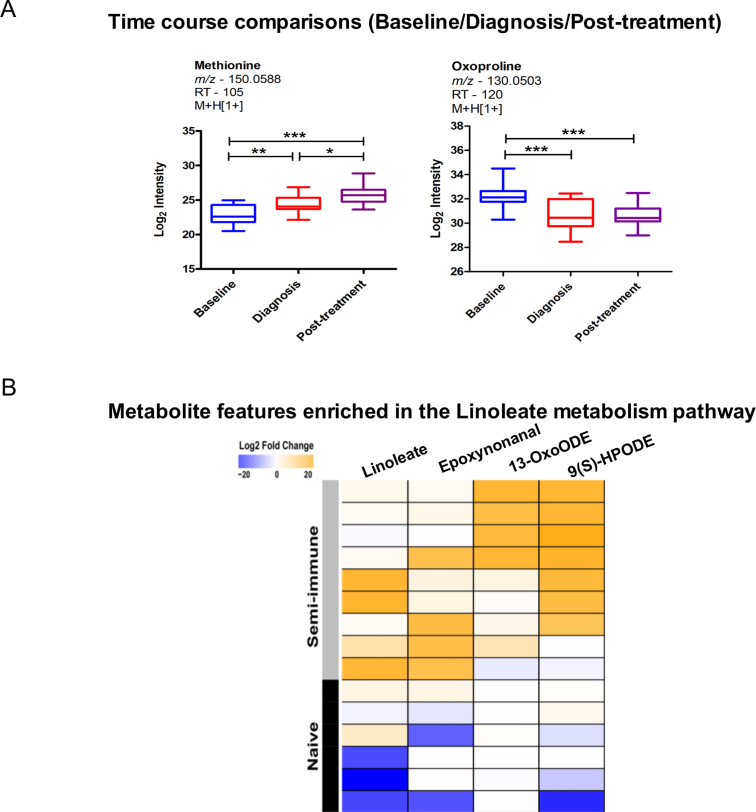
**Differential metabolite abundance.***A*, Boxplots for methionine and oxoproline across the three time-points of the *P. vivax* CHMI trial. Differential abundance was calculated with repeated measures ANOVA, and Tukey’s multiple comparisons test, between baseline, diagnosis and post-treatment time points. Significance levels are shown as * , p < 0.05; **, p < 0.01; ***, p < 0.001. *B*, Heat map of differentially abundant metabolite features enriched in the linoleate metabolism pathway. Blue to orange scale indicate log2 fold change of diagnosis relative to baseline levels. Tentative annotation from the *mummichog* software, including multiple adducts.

This CHMI study using *P. vivax* was monitored as a time course: baseline was taken as 2 days prior to infection; parasites were detected in the subjects’ blood around 10 days after infection, and treatment was started on this same day of diagnosis. The treatment included three doses of chloroquine and 14 days of primaquine. Plasma samples from three time-points, baseline, diagnosis and post-treatment, were analyzed by untargeted high-resolution metabolomics. This resulted in detection of over 8000 metabolite features. After filtering for missing values, 4236 were retained for statistical analysis. The abundance of 964 metabolite features was significantly altered across the three time-points (p < 0.05; 0 at FDR < 0.1; Fig. 1A-B), irrespective of the immune status of subjects, while one-way hierarchical clustering revealed high variability among the subjects (Fig. 1B). To evaluate the metabolic pathways underlying these significant metabolite features, we used *mummichog*, a software specifically designed for untargeted metabolomics [25], which has gained increasing popularity [34], [39], [40]. The results indicate that significant features are enriched for pathways involved in amino acid metabolism, carbohydrate metabolism, energy metabolism, lipid metabolism and nucleotide metabolism (Fig. 1C). Examples of these significant metabolites include carnitine (Fig. 1D), methionine and oxoproline (Supplementary Figure 1A).

The metabolites associated to time-points suggest that a greater metabolic shift was seen post-treatment than at the time of diagnosis (Fig. 1B). Indeed, when each time-point of diagnosis or post-treatment was compared to baseline, the greatest effects in the plasma metabolome of all subjects occurred post-treatment (Fig. 1E). This observation remained true when the naïve group and semi-immune group were analyzed separately (Fig. 1E, F-G). Specific metabolites, however, appeared to be significant in each of the groups. For the naïve subjects, these include tryptophan, kynurenine and glutamine (Fig. 1H, Supplementary Table 1). For the semi-immune subjects, these include glutamate, N-acetyl-leucine and pantothenate (Fig. 1H, Supplementary Table 1). Overall, these data suggest that the treatment drugs had a major effect on the host metabolome. In addition, the infection also induced metabolic changes in peripheral blood by the time of diagnosis, which was more pronounced in the semi-immune group (Fig. 1E, G).

### 3.2 Naïve and semi-immune subjects exhibit distinct metabolic profiles in plasma

To investigate the metabolomic difference between naïve and semi-immune subjects, we compared the two groups at baseline, diagnosis normalized to baseline and post-treatment normalized to baseline. At diagnosis/baseline, 126 metabolite features differed significantly between the two groups (p < 0.05), while greater numbers were seen at baseline and post-treatment (Fig. 2A, B). This does not necessarily indicate that the baseline difference was greater, because it was removed in the comparisons after infection by subtracting the baseline values. The differential metabolites at three time-points are given in Supplementary Table 1. A few examples include uric acid and indole 3-acetaldehyde (Fig. 2C).

We compared the significant pathways over the time course in each group (Fig. 2D, left), and between the two groups at each time point (Fig. 2D, right). To be consistent in all six analyses, the 200 most significant metabolite features were used for pathway test in each case. In the time course analysis, it appears that most pathways were driven by changes relative to the naïve group, most notably methionine and cysteine metabolism and glycine, serine, alanine and threonine metabolism. The semi-immune group exhibited a significant change in urea cycle/amino group metabolism, linoleate metabolism and arginine and proline metabolism. When the two groups were compared to each other, few pathways showed significant enrichment, mostly at post-treatment. Linoleate metabolism, however, reached significance again at the time of diagnosis (Fig. 2D, Supplementary Fig. S1B). Interestingly, the 3 major metabolite features related to linoleate are derived from lipid peroxidation reactions. Epoxynonanal is an oxidation product that is likely to be derived from the decomposition of the lipid hydroperoxide 9(s)-HPODE. The lipid peroxide can be formed either enzymatically through the action of lipoxygenase or through non-specific lipid peroxidation and the 13-OxoODE is known to also be derived from the enzymatic decomposition of lipid peroxides [41]. This together with significant changes in the lipid metabolites prostaglandin and eicosapentaenoic acid (Supplementary Table 1) indicates a difference in inflammatory response at the time of diagnosis between two groups. Interestingly, although many metabolite features differed at baseline, they did not significantly match known metabolic pathways (Fig. 2D).

The difference in urea cycle/amino group metabolism and arginine and proline metabolism is exemplified by glutamine (Fig. 2E). Glutamine is converted to glutamate by glutaminase (GLS), and is a major precursor for the *de novo* synthesis of arginine in humans [42]. Both arginine and glutamate are involved in urea cycle. Arginine is depleted during *P. vivax* malaria, and this reduction is associated with endothelial dysfunction [43]. The level of glutamine was examined in *P. falciparum* malaria before with varying conclusions [44], [45]. Our data indicate that glutamine levels remained relatively stable in semi-immune subjects, but decreased after infection in the naïve subjects (Fig. 2E). Glutamine levels exhibited a clear and inverse correlation with GLS expression level assayed by RNAseq (Fig. 2F).

### 3.3 Transcriptomic programs differentiate naïve and semi-immune subjects

The blood transcriptomics of this *P. vivax* CHMI study was obtained by RNAseq using mRNA from whole blood at baseline and at diagnosis. The initial analysis was described previously [37]. Consistent with the earlier publication, significant changes in gene expression occurred after infection, but no apparent difference was seen between naïve and semi-immune subjects at gene-level analysis (Fig. 3A). The recently developed new tool, Blood Transcription Modules (BTM), demonstrated higher sensitivity of capturing immunological events from blood transcriptomics [26]. Using BTM with the well-established GSEA software indeed shows significantly differential gene modules between the two groups. For example, a neutrophil gene module was significantly higher in semi-immune compared to naïve subjects (Fig. 3B).

A summary of significantly enriched BTMs is shown in Fig. 3C. Both naïve and semi-immune groups exhibited up-regulation of type I interferon response and dendritic cell activation, and decrease of neutrophil signals after infection (Fig. 3C, left three columns, Fig. 3D). Direct comparison of naïve and semi-immune subjects revealed detailed difference in their immune responses (Fig. 3C, right two columns). While the semi-immune subjects showed a higher level of B lymphocytes at baseline, at the time of diagnosis, they were highly enriched for modules corresponding to an inflammatory response mediated by myeloid cells. This is consistent with the inflammatory lipids on linoleate pathway at diagnosis from plasma metabolomics data (Fig. 2D, Supplementary Figure 1B). The T cell modules showed a lower level of signals in these subjects, but this can be attributed to homeostasis that offsets the higher level of myeloid cells. Both volunteer groups had decreased number of neutrophils after infection, but the neutrophils in the semi-immune group still significantly outnumbered those in the naïve group (Fig. 3C, D). The semi-immune group also displayed higher activity in other processes, such as cell cycle and blood coagulation (Fig. 3C, D). Taken together, these data indicate that the semi-immune group launched a stronger innate activation at the time of diagnosis, which then contributed to their improved control of symptoms after *P. vivax* infection.

### 3.4 Infection with *Plasmodium vivax* induces concerted metabolic and transcriptional responses

Although the transcriptomics was measured from circulating immune cells and metabolites from plasma, the above data suggest that they are not isolated from each other. Recent literature also indicates that metabolites are integral signals in immune responses [16], [17], [46]. We therefore set out to investigate the associations between transcriptomics and metabolomics data. We adopt here a method recently published by our group [16]. This method combines meaningful dimension reduction with PLS regression. The transcriptomics data were collapsed into BTM clusters, and metabolomics data were collapsed into metabolite clusters. The PLS regression accounts for different variance structures of different technologies. The statistical significance of those associations was evaluated by permutation [16]. This has the added benefit on statistical power because only consistent signals in both data types will reach significance.

**Fig. S2:**
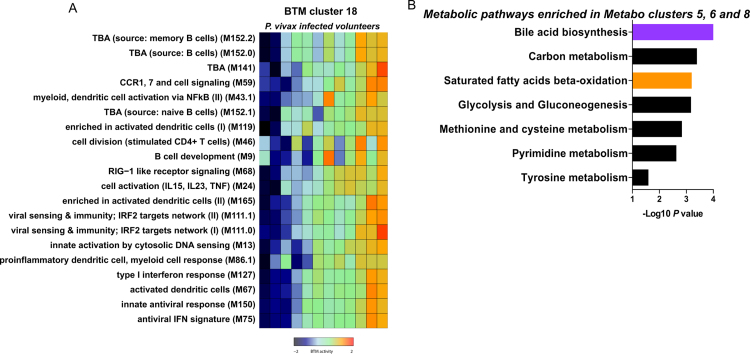
**Example of BTM and Metabolite clusters relative to**Fig. 4. *A,* Heat map of BTM’s activity for each *P. vivax* infected subject, included in BTM cluster 18. *B*, Combined metabolic pathways enriched in Metabolite clusters 5 (black bars), 6 (purple bar) and 8 (orange bar). *Mummichog* software was used to evaluate metabolic pathways.

At baseline, only one significant association between transcriptomics and metabolomics was detected (Fig. 4A), not surprisingly, because of the small cohort and the contribution of transcriptomics and metabolomics coming from different compartments (cells and plasma, respectively). However, in stark contrast, infection with *P. vivax* induced many significant associations between the two data types (Fig. 4B, 26 edges with p < 0.001, 88 edges with p < 0.05). These associations center on three transcriptomic hubs (BTM clusters): IFN signature and innate cell activation gene modules, chemokines and T cell signaling, and platelet and complement activation (Fig. 4B). As type I IFN response is important in the transcriptomic signature (Fig. 3C, D), it's expected to be concordant with several other innate pathways (Supplementary Figure 2A). The hub on Chemokines and T cell signaling (BTM cluster 23) is further listed in Fig. 4C, and member genes in Fig. 4D. This overlaps with the inflammatory response and myeloid cell signature in Fig. 3C (RA, WNT, CSF receptors and network), which differed between the naïve and semi-immune groups. Although other gene modules share a similar profile, not all modules are significant in Fig. 3C, as different statistics is applied here to identify the most association with metabolites. One of the metabolite clusters associated with this hub is exemplified in Fig. 4E, and consisted of 205 metabolite features of similar profiles. The members in this cluster are related to amino acid metabolism (Fig. 4F), and the examples of tyrosine and serine are shown in Fig. 4G. The associations between amino acids (tyrosine, serine and glutamate) and M38 members *CXCR4* and *IL8* are further illustrated in Fig. 4H.

### 3.5 Metabolomic confirmation of platelet involvement in response to *P. vivax* infection

The other significant transcriptomic hub relates to platelet and complement activation (Fig. 4B). This cluster consists of genes mostly related to platelet activation, blood coagulation and SRF (serum response factor) signaling (Fig. 5A). Their expression level is higher in the semi-immune group at the time of diagnosis (Fig. 5B), though both naïve and semi-immune groups exhibited platelet depletion after *P. vivax* infection (Fig. 5C), consistent with previous literature [47]. Associated with this hub are many metabolite clusters encompassing 881 features (p < 0.05 Figs. 4B, 5D). Platelets are now increasingly recognized as immune cells [48], [49]. Platelets mediate the agglutination of *P. falciparum*-infected erythrocytes [50], and play a protective role by killing intraerythrocytic *P. falciparum* parasites [51]. However, *P. vivax* infected cells do not seem to adhere to platelets [52], suggesting distinct platelet-mediated effector mechanisms [53]. The quantitative difference in platelet signals appears to be a key part in the different responses to *P. vivax* infection between the naïve and semi-immune groups.

The significant association between platelet activation modules and metabolomics data (Fig. 5D) raised the possibility that the plasma metabolomics was strongly influenced by platelets. This is possible because platelets remain in the plasma layer after whole blood is centrifuged and processed. To test this hypothesis, we assessed platelets from healthy donors and performed metabolomics analysis on the same LC-MS platform. Among the 881 metabolite features associated to BTM cluster 8 (p < 0.05; Fig. 5E), 309 were found in the platelet metabolome (Fig. 5F). We then compared each of these metabolite clusters against the 200 most abundant *m/z* peaks in platelets, and they were clearly enriched in platelet metabolites (Fig. 5G). The enrichment p-value for metabolites in cluster 3 was under 10^−23^ (hypergeometric test). These data confirm that the plasma metabolomics captured a strong signal from platelets, and our integration method was successful in identifying this concerted event between blood transcriptomics and plasma metabolomics. Taken together, these data indicate that the *P. vivax* infection triggered a broad array of concerted molecular programs in systemic metabolites and blood cells.

## 4 Discussion

Clinical tolerance to re-infections with *Plasmodium* has been well documented in endemic regions for malaria [54]. This phenomenon is likely driven by the coordinated activity of multiple biological processes, which are not completely understood. Oxidative stress is reportedly part of the infection and pathogenesis of malaria [55]. This is supported by the many amino acid and lipid pathways identified in this study. Moreover, redox is a key part of immune response in both pathogen suppression and host signaling [56], [57]. Recent studies show that combined metabolomics and transcriptomics analyses provide sensitive approaches to link oxidative stress to biologic responses [58]. Here we demonstrate that, compared to naïve subjects, those with previous history of *P. vivax* malaria exhibited distinct plasma metabolic signatures during a CHMI trial. Notably, naïve subjects exhibited greater perturbations on the plasma metabolome in a time course analyses. However, metabolic signatures from semi-immune subjects showed a higher discriminative power, suggesting a more coordinated response after repeated exposure to *P. vivax*. The semi-immune subjects in this study developed minor to no symptoms [8], and complete tolerance often requires several episodes of malaria [54]. Thus, a short-lived immunological memory might account for differences between clinical outcomes [54]. These subjects were recruited from different regions in Colombia and may exhibit slightly differing life-styles and while these could influence the metabolic phenotypes it is unlikely they make a major contribution to the differences in the patient cohorts reported here. Nevertheless, our data supports the hypothesis that clinical tolerance to *P. vivax* results in a distinct metabolomic profile, especially linoleate metabolism.

During the CHMI trial, several amino acids and related metabolites displayed differential kinetics, including glutamine, kynurenine and tryptophan. Kynurenine's abundance increased with concomitant depletion of tryptophan in plasma of naïve subjects after *P. vivax* infection but returned to baseline levels after treatment. Both tryptophan and kynurenine's abundance remained unaltered in semi-immune subjects (Fig. 1H). The conversion of tryptophan to kynurenine is catalyzed by indoleamine 2,3 dioxygenase (IDO), and the inhibition of IDO was shown to protect mice from lethal malaria parasite infection [59]. Therefore, tryptophan metabolism may play a role in the pathogenesis of *P. vivax* malaria. Kynurenine contributes to arterial-vessel relaxation during malarial inflammation in mice [60], and could also be involved with endothelial dysfunction and hypotension in severely ill *P. vivax* patients. Of note, a higher proportion of naïve subjects had severe headaches [8]. While caused by *P. falciparum*, children with cerebral malaria were shown to possess elevated levels of kynurenine-derived metabolites in their cerebrospinal fluid [61]. Moreover, inhibition of this pathway prolongs survival in a mouse model of cerebral malaria [62]. We hypothesize that this pathway may be a target to reduce symptoms of *P. vivax* malaria.

The semi-immune group also differed from the naïve group in inflammatory lipids related to the linoleate pathway. These lipid mediators constitute a complex bioactive network with potent inflammatory and regulatory properties and interestingly decomposition products from lipoxygenase derived lipid peroxides [63]. In addition to the lipid peroxidation products produced by the enzymatic action of lipoxygenases, cyclooxygenase products are also produced. For example, prostaglandin E2 (PGE2) inhibits type I interferon response to *Mycobacterium tuberculosis*, which is crucial for protection [64]. *P. vivax* infection induces the transcriptional activity of type I interferon pathway [37], while PGE2 levels are reduced during severe *P. vivax* malaria [65]. Our metabolite data corroborate the transcriptomic response: at the time of malaria diagnosis, the transcriptional profiles of semi-immune subjects mainly reflect a response mediated by myeloid cells when compared to naïve subjects. Thus, the tolerance to clinical symptoms might involve more than the memory developed by lymphocytes, and may also rely on trained innate immune cells. This concept emerged from observations that innate immunity can be crucial for re-infections [66]. Indeed, trained immunity involves epigenetic and metabolic reprogramming of innate immune cells [66] and is induced by *P. falciparum* infection [67]. Our data suggest that metabolic reprogramming of myeloid cells could have a substantial effect on the development of clinical tolerance to re-infection with *P. vivax*, in which platelets play a significant role. Recent work demonstrated that repeated exposures to *P. vivax* results in higher frequencies of classical memory B cells, reduction of atypical memory B cells, and increased levels of *P. vivax*-specific IgG [68]. Moreover, women exposed to *P. vivax* exhibited a higher proportion of atypical memory B cells [69]. Thus, the extensive associations with BTMs related to B cells at baseline for semi-immune, but not naïve subjects, indicate that clinical tolerance to *P. vivax* malaria is accompanied by increased circulation of memory B cells.

To our knowledge, this is the first study to integrate host metabolomics and transcriptomics in the context of human malaria research. The major limitation of this study is the small cohort size. However, this will be a common challenge as we move towards precision medicine approaches to big data. It would be useful to test the hypotheses in a separate cohort, a larger population or appropriate animal models. Without any of those, one way to address the challenge is the integration of orthogonal and intensive data points – the integration of metabolomics and transcriptomics here enhanced the statistical power. Because of the limitation of sample size, little significant association was found between the orthogonal datasets at baseline. However, stronger signals elicited by the infection pinpointed to a concerted network, hinging on gene modules related to platelet activation, type I interferon and innate immunity, and for chemokines and T cell signaling. Among the associated metabolic pathways, linoleate and glycerophospholipid metabolism were also associated with the transcriptional activity of innate immunity, T and B cells in response to a live attenuated viral vaccine in humans [16]. By measuring the platelet metabolome separately, we were able to confirm that the plasma metabolites associated with platelet activation genes in blood cells were indeed highly enriched in platelets. Overall, these results indicate that the immune response to *P. vivax* infection is tightly associated with host metabolic responses. A better understanding of their interplay will be useful for designing novel therapeutic interventions and vaccines.
